# Self organising maps for visualising and modelling

**DOI:** 10.1186/1752-153X-6-S2-S1

**Published:** 2012-05-02

**Authors:** Richard G Brereton

**Affiliations:** 1 School of Chemistry, University of Bristol, Cantocks Close, Bristol BS8 1TS, UK; 2 Brereton Consultancy, New Bond House, Bond Street, Bristol BS2 9AQ, UK

## Abstract

The paper describes the motivation of SOMs (Self Organising Maps) and how they are generally more accessible due to the wider available modern, more powerful, cost-effective computers. Their advantages compared to Principal Components Analysis and Partial Least Squares are discussed. These allow application to non-linear data, are not so dependent on least squares solutions, normality of errors and less influenced by outliers. In addition there are a wide variety of intuitive methods for visualisation that allow full use of the map space. Modern problems in analytical chemistry include applications to cultural heritage studies, environmental, metabolomic and biological problems result in complex datasets. Methods for visualising maps are described including best matching units, hit histograms, unified distance matrices and component planes. Supervised SOMs for classification including multifactor data and variable selection are discussed as is their use in Quality Control. The paper is illustrated using four case studies, namely the Near Infrared of food, the thermal analysis of polymers, metabolomic analysis of saliva using NMR, and on-line HPLC for pharmaceutical process monitoring.

## Introduction

The analysis of multivariate data from laboratory instruments using computational methods has been a subject of academic pursuit since the 1970s, often loosely called chemometrics [1-22]. The early pioneers of the 1970s were primarily analytical chemists such as Bruce Kowalski and Luc Massart, although Svante Wold was on the interface of analytical and organic chemistry. Methods such as PCA (Principal Components Analysis) [22-27] and PLS (Partial Least Squares) [28-34] were developed and widespread applications reported in the literature. Much of the early applications involved problems such as Near Infrared Calibration, deconvolution of Gas Chromatography Mass Spectrometry or High Performance Liquid Chromatography signals and determining of components in mixtures using Ultraviolet Visible Spectroscopy of mixtures. These types of datasets slowly became widespread with the spread of computerised instruments in laboratories. The spread of these methods was particularly pronounced as from the mid 1990s when user friendly software became widely available.

These pioneering methods were first primarily developed as applied to traditional analytical chemistry. Data had several features that made these methods suitable. The first is that most data was linear and additive. Often great efforts were made to ensure that signals were within a linear region as defined by the Beer-Lambert law, and also above the limit of detection. The second is that residuals (or noise) could often be approximated by a normal distribution. Sometimes if noise was shown to be heteroscedastic, data was scaled to overcome this limitation. The third was that on the whole the number of samples was limited, typical published reports in the early literature involved between 20 and 50 samples. A fourth feature was that most early chemometrics involved predictive modelling. The aim was to measure a parameter, such as the concentration of a compound in a series of mixture spectra, to a given degree of accuracy : usually the concentration of a series of reference samples was known accurately and the aim, would be to determine this using, for example, mixture spectroscopy. Finally, desktop computing power was limited and so algorithms such as NIPALS (nonlinear iterative partial least squares) [35] and cross-validation [36] were developed especially to be efficient both in cpu time and memory useage.

Over the years, the software packages first marketed in the 1980s or early 1990s, such as Infometrix's Pirouette [37], Camo's Unscrambler [38], Umetrics SIMCA [39] and Eigenvector's PLS toolbox [40] amongst many others, were improved, but in most cases this has been in the interface and user-friendliness, keeping up to date with modern expectations for computer users. The underlying philosophy of PCA and PLS has hardly changed. A lot of effort is required to update software and it is labour intensive and risky to enter new markets.

However much has changed during the last few decades.

1. Computers have become more powerful. Moore's law originally suggested that computing power per unit cost doubles every year [41]. Taking a more conservative estimate of a doubling every 18 months, this means that in the 30 years since 1981, computing power has improved around 2^20^ or around a million fold. Put another way, a calculation that took a week on a typical desktop in 1981 would take around 0.6 s in 2011 for equivalent unit cost . Although these are approximations, many readers can see how kilobytes of memory in 1981 translate into gigabytes in 2011. The ZX80 marketed in the UK in 1980 for £99.95 consisted of 4 kb memory. An equivalent price taking inflation into account in 2010 is £320 [42]. Certainly typical PCs could be purchased with between 3 Gb and 4 Gb memory in 2010, with prices below £400, so this rate of increase (of memory, speed and discspace) per unit cost holds. Therefore we can perform calculations on our desks that might have required expensive access to institutional mainframes many years ago. Hence relatively computer intensive methods are now feasible.

2. The capability of analytical instruments has increased dramatically. Using autoinjectors and self programmable instruments, rapid sample throughput can be achieved. In addition most instruments are much more stable so require less manual tuning, hence many samples can be throughput. In some applications such as NMR, the advance in automated data collection is dramatic. In addition automating methods for data storage and retrieval mean less manual interpretation of spectra and chromatograms. Hence the amount of the data to be studied has increased substantially.

3. Finally, whereas traditional analytical chemistry exists and will continue to exist, the range of problems being routinely study in analytical laboratories has changed. Biology, medicine, cultural heritage, archaeology and environment are all legitimate and ever increasing areas that the analytical chemist can contribute to. These problems are not necessarily linear ones, we do not for example expect the amount of a compound found in a cultural artefact to be linearly related to its age, nor do we expect the concentration of a biomarker to be linearly related to the progression of a disease. The vast majority of datasets fail normality tests and so cannot be safely analysed using statistics dependent on assumptions of normality. Outliers are quite common and have a potentially strong influence using least squares methods. Whereas traditional linear approaches can be adapted to these situations, the adaptations are often clumsy, and most users of packaged software are unaware of these.

Hence modern developments pose the need for different approaches to those traditionally employed by chemometricians. Over several decades there have been developed approaches for machine learning. The originators were primarily computer scientists and had access to powerful institutional computers. The early descriptions were somewhat theoretical and mainly applied to well established reference datasets, but have spread rapidly in areas such as economics, medicine and biology. They were developed primarily for data mining.

Self Organising Maps (SOMs) were first proposed by the Finnish computer scientist, Teuvo Kohonen [43-45]. The original use was primarily to visualise data. There is a slow but steady increase in their useage within analytical chemistry [46-51]. The key barriers to use of SOMs are as follows.

1. There are less user-friendly packages around compared to traditional chemometrics approaches. Many chemists want simple plug in packages that require limited or no knowledge of programming, unlike, for example, bio-informatics experts who are usually happy to edit or adapt code. This also means that advocates of niche chemometrics packaged software do not have these yet as part of their repertoire and therefore are unlikely to advocate their useage.

2. The packages are computationally intense, although as discussed above, this is no longer a serious barrier. However many chemists read literature of a few years ago, and old habits die hard so are not familiar with their potential.

3. Often specialist expertise is required at the moment, unlike for PCA where there are innumerable cheap or free facilities. This expertise is not always available in laboratories, and can be costly to hire.

4. The results are not reproducible, differing each time. This should not be a serious barrier, and one approach is to repeat the SOM many times over to reach a consensus or stable solution.

5. SOMs have much potential for graphical output. Up to recently it has been costly to publish papers in colour, so the potential for data display was somewhat limited in print journals. Many scientists follow the lead of existing published work and as such did not encounter sufficient publications that fully illustrated their potential. With the growth of on-line publishing and cheaper colour printing there are many more recent examples, however, it takes time for new ideas to filter down the line.

6. Finally, up to recently SOMs have been less adapted to quantitative useage, which is important for their acceptance to most chemists. However there are now starting to be published new adaptations.

Hence many of the barriers to use of SOMs as a tool for the chemist are gradually disappearing. The desire of many laboratory based chemists to analyse data themselves still poses a problem: in areas such as biology and medicine it is usual for there to be separate data analysis groups, so novel computational approaches can be adopted much faster and do not need to wait for commercial package developers.

In this paper we will describe the basis of SOMs and their most recent developments, illustrated by case studies as applicable to complex chemical datasets.

## Case studies

Details of the case studies have already been published elsewhere. In this section we provide basic details, and refer the reader to the in-depth references for further experimental details. It is not necessary to understand the methods for data preparation to appreciate this paper, although it is crucial these are performed correctly for meaningful analysis. The reader is referred to the original papers for more details on preprocessing including the motivation behind choice of methods..

### Case study 1 : NIR of food

This dataset [3,50] consists of 72 Near Infrared spectra of

1. 18 samples of Corn Oils

2. 30 samples of Olive Oils

3. 16 samples of Safflower Oils

4. 8 samples of Corn Margarines.

The data has been preprocessed using Multiplicative Scatter Correction, wavelength selection and mean centring prior to data analysis. The aim is to classify samples using NIR spectra into one of four groups.

### Case study 2 : thermal profiles of polymers

This dataset consists of the thermal profiles of 293 samples, involving monitoring the change in physical properties as they are heated. The polymers can be divided into two types namely class A of 92 amorphous polymers and class B of 201 semi-crystalline polymers. In turn, each type consisted of nine different groups as listed in Table 1. More detail is discussed elsewhere [3,51-54].

**Table 1:** Samples for case study 2

Type			Group		
Amorphous	A	92	Polystyrene (PS)	A	35
			Acrylonitril- Butadiene-Styrene (ABS)	B	47
			Polycarbonate (PCarb)	C	10

Semi-crystalline	B	201	Low Density Polyethylene (LDPE)	D	56
			Polypropylene (PP)	E	45
			High Density Polyethylene (HDPE)	F	30
			Polyamide6 (PA6)	G	20
			Polybutylene Terephthalate (PBT)	H	10
			Polyethylene Terephthalate (PET)	I	40

The aim is to determine which group a polymer belongs to from their thermal properties. There is also a secondary structure to the samples in that the groups can be divided into two types.

### Case study 3 : NMR metabolic profiling

This case study consists of 96 NMR spectra of saliva extracts, involving a 2 x 16 x 3 design, where there are 2 treatments (mouthwash or control), 16 donors and 3 sampling days [3,55,56]. The samples can therefore be classified in one of three ways. For example, there are 6 samples from each donor, 3 of which are treated and 3 not, each taken on different sampling days. Therefore we may expect some distinct groupings due to donor as well as treatment, each grouping influenced by different biomarkers (or regions of the spectrum – called variables). Sampling day is a dummy factor in that it should have no significant influence on the spectra.

The NMR spectra are preprocessed using baseline correction, bucketed into regions and then scaled by square rooting and centring. The aims are to determine whether there are groups in the data due to individuality and treatment, and what parts of the spectra are responsible for these distinctions, which give a clue as to which biomarkers are significant.

### Case study 4 : on-line HPLC for process monitoring

This dataset involves monitoring the first stage of a three stage continuous process. More details have been described elsewhere [57-59]. 309 samples were recorded using on-line HPLC over a period of 105.11 hours. 12 peaks were chosen from the chromatograms and their areas were recorded after baseline correction, square rooted and summed to a constant total in each chromatogram and used to monitor the process.

Sample numbers 63 to 92 (21.71h to 32.17h) were defined as the NOC (Normal Operating Conditions) region, that is the part of the process considered to be “in control” or typical of the process. For subsequent samples the aim is to see whether they have characteristics of the in control region or not. If not, it is diagnostic of a problem with the process, as the expected relative peak areas have deviated from the norm.

## Basic SOM algorithm

The basic SOM algorithm has been described in detail elsewhere [3,51] and we will focus just on the main definitions and steps in this section without mathematical detail. The algorithm described in this section is the unsupervised and original one.

### Maps, component planes and best matching units

**Figure 1:**
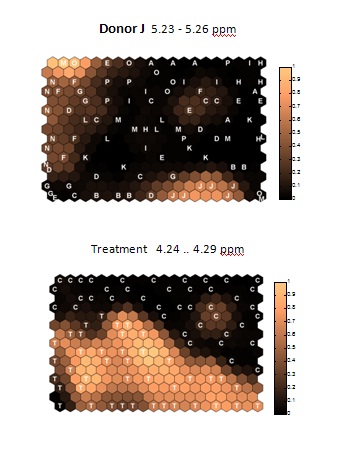
Principle of SOMS (a) A 6 x 5 map (b) Component Planes (c) Best Matching Units.

Figure 1 illustrates a schematic of a SOM. A map is made up of cells (or units). In the case illustrated the map consists of 30 cells, or a 6 x 5 map. Usually we visualise just the front of the map, which relates to the samples or objects in the dataset. However behind the cells are component planes. These correspond to the variables used to create the map. If there are for example 100 spectral wavelengths, there will be 100 component planes. These "hidden" layers correspond to analytical measurements such as spectra or chromatograms. Behind each cell is an array equivalent to a spectrum, if there are 30 cells, there will be 30 such arrays. In the case illustrated there are just 3 variables, so three component planes. Each sample in the training set has a corresponding BMU (or best matching unit) in the map. This is the cell that a sample is most similar to. Ideally the samples in the training set are spread around the map. In the case illustrated there are 10 samples, 6 from a blue group and 4 from a brown group. They slot into 10 corresponding cells.

### Size of map

The size of the map can be controlled, and usually if it is represented as a grid as in Figure 1, the number of horizontal and vertical cells is different. A good rule is to make the grid about three times the number of samples, this is to allow the samples to spread around. Occasionally more than one sample fits into a single cell. This may be because the samples are extremely similar or that the number of cells in the map is small relative to the number of samples.

### Iterative development of map

Usually a map is usually developed from a random starting point. A starting map consists of a set of component planes, that is a randomly generated vector is associated with each unit or cell in the map.

**Figure 2:**
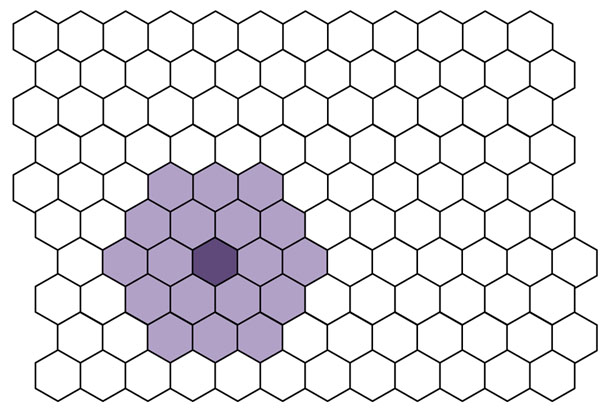
A BMU and its neighbours.

The next step is to randomly pick a sample from the dataset and identify which cell is its BMU. Then the cells close to the BMU are identified. The number of cells is given by a parameter called the neighbourhood width, a large neighbourhood width implies that many cells are identified, and small neighbourhood width, only a few. The process involves making the component planes of the neighbouring cells a little more similar to the central (BMU). Figure 2 illustrates a BMU and its neighbours.

This procedure is continued many times, often several thousands of times, each time a fresh sample is chosen. The number of iterations should be many times the number of samples to ensure each sample is chosen several times. The chance that a sample is never chosen is [(*N-*1)/*N*]*^T^* where *N* is the number of samples, and *T* the number of iterations. If there are 100 samples, this chance is 4 x 10^-5^ for 1,000 iterations and 2 x 10^-44^ for 10,000 iterations. The age of the universe is around 4 x 10^17^ seconds, so even if SOMs were calculated continuously on a dataset from the time of the big bang, and 10,000 iterations could be performed on a superfast computer in 1 s, a sample would still only have a chance of one thousand, million, million, million, millionth of being missed out after 10,000 iterations, rather like some unimaginable quantum mechanical event of walking through a wall. For 1,000 iterations there is an extremely remote chance a sample is left out. If however there are 500 samples in the training set, and only 1,000 iterations are used, the chance that a sample is left out is 0.13 or 13%. Hence the number of iterations should in part be related to the number of samples, although if a sample is left out altogether it still can be fitted into the map, but has no influence on training.

As the number of iterations increase, the region of cells that is adjusted around the BMU is reduced, and the amount of adjustment (often called the learning rate) also reduces. This means that the maps start to stabilise. The more the iterations the more computationally intense the SOM, and sometimes it is possible to reach an acceptable solution fast. Most SOMs are developed using a random starting point, although there are modifications that allow an initial map that reduces the number of iterations by basing it on the pattern of the samples, e.g, as obtained via PCA.

### Variables

The variables that are used to describe the map usually are the raw measurements, such as spectral intensities or chromatographic peaks. Under such circumstances it is possible to interpret the component planes to provide chemical insight. However sometimes the number of variables is large, and it can be time consuming to use all the original variables, especially if some are primarily noise. Hence an alternative is use functions of the variables such as Principal Components.

### Geometry of SOMs

The simplest geometry is as a rectangular map. The rectangle refers to the arrangement of cells and not the shape of the cells. Often the cells are represented as hexagons, as we will do in this paper, but can be represented by squares.

However there is no obligation to restrict the maps to rectangular ones, and circular, cylindrical or spherical maps can be visualised. One problem of rectangular maps is that samples at the edges tend to be farther away from other groups of samples to those in the middle, that may have many more neighbours. Some datasets do, indeed, have extreme groups of samples, and so the rectangular approach is the most appropriate. But in other cases there may well not be any reason to separate out samples that are on the extreme edges and so a spherical or cylindrical representation is more appropriate. The trouble with the latter representations are that they are harder to visualise on paper.

A representation though can be retained in a computer, and the aim is not so much to present a graph to the user but to use the co-ordinates of samples to show which are most similar, then having other geometries could be worthwhile. In this paper we will restrict representations primarily to the most common type of map, which is rectangular, and use hexagonal cells.

## Visualising SOMs

### Best matching units

**Figure 3:**
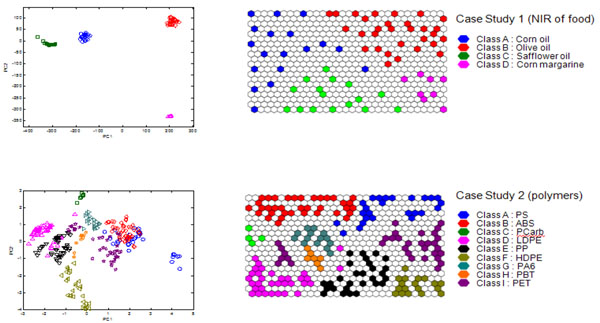
Plot of scores of the (left) first two PCs and (right) BMUs of (top) case study 1 – NIR of four foods and (bottom) thermal profiles of nine groups of polymers.

The most basic approach is to represent the samples on the map is via their BMUs (Best Matching Units). Figure 3 represents the BMUs of 30 x 20 maps obtained from case studies 1 and 2, together with the scores of the first two PCs. For case study 1 (NIR of food) the number of cells (600) far exceeds the number of samples (72) and as such the samples are well separated. The map of case study 2 is somewhat more crowded with a ratio of cells : samples of around 2 : 1 and as well will see in the section on 'Hit histograms', some cells are the BMUs of more than one sample. However still the samples are reasonably well spread out.

The SOM map visualisation has some advantages over the corresponding PC scores plot. First of all the full space is used efficiently. In PC scores plots sometimes there can be crowded as there may be many samples that have to be represented in scores space. In other cases, the space is used inefficiently with lots of blank space. The second advantage is that there is no need to choose which PCs are to be used for visualisation. Third, there are many more options for graphical representation as discussed in this paper.

Figure 3 represents samples projected onto the scores of the first two PCs for case studies 1 (NIR of food) and 2 (polymers), with a corresponding SOM representation together with BMUs. For case study 1, although the groups are tightly clustered, the majority of the PC space is wasted, and basically meaningless as there are no samples and no information available for the "empty" regions. The groups are so tightly clustered that we cannot see any structure within the groups. For case study 2, again much of the PCspace is wasted, and the groups overlap considerably, the symbols becoming quite crowded and hard to distinguish. These problems are no longer disadvantages in the SOMs. In addition there are a large number of ways of shading and representing symbols.

People that are not trained data analysis experts often find SOMs easier to understand and interpret, a map being more intuitive than a scores plot or complex graph.

Note that BMUs can also be used for predicting the provenance of unknown samples, or a test set, simply by seeing which places in the map they fit into. This concept of having a "board" where unknown samples are slotted into is also intuitively easier for most users to understand than predicted positions of points on a graph.

### Hit histograms

**Figure 4:**
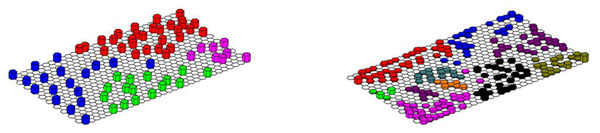
Hit histograms for (a) left : case study 1 (b) right : case study 2.

A hit histogram can be consider as a three dimensional projection of the BMU map. The hit histograms for case studies 1 and 2 are illustrated in Figure 4. In each cell that corresponds to a BMU for the training set, there is a vertical bar that represents the hits. Each sample in the training set will be represented on the map.

For case study 1, there are 72 samples and each hits a different cell in the map, so there are 72 vertical bars. The map is of size 30 x 20, or consists of 600 cells, so there is plenty of room for the samples to spread around. We notice that for case study 2, there are 293 samples, or roughly half the number of samples compared to the map. Some samples have the same cell for their BMU, for example, there are several samples of HDPE - see Figure 3 for interpretation of symbols - on the bottom right corner that overlap with each other. This is not clear on the BMU map, which is clarified in the hit histogram.

If there is more than one sample associated with an individual BMU, then either this is tolerated or a map with more cells can be generated. The problem with maps that have more cells is that they are slower to train. For case study 2, most people would tolerate a small level of overlap.

### Class map

**Figure 5:**
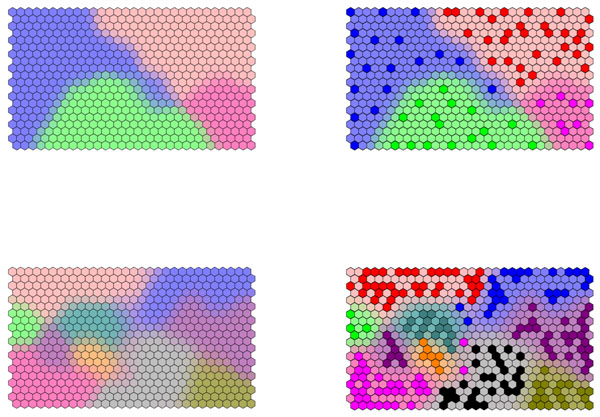
Class diagrams of (top) case study 1 and (bottom) case study 2. BMUs are indicated in the right hand column.

If samples fall into groups, or classes, this additional information can be used to shade the background on the SOM. A cell is shaded in the colour of its closest BMU. If more than one BMU is equidistant from the cell, it is shaded in a combination of colours, according to how many BMUs from each group it is closest two. For example if a cell is closest to two BMUs from class A and one from class B, it is shaded in 2/3 the colour of class A and 1/3 of class B. The class diagrams for case studies 1 and 2 are illustrated in Figure 5. In the right column, the BMUs are also presented.

**Figure 6:**
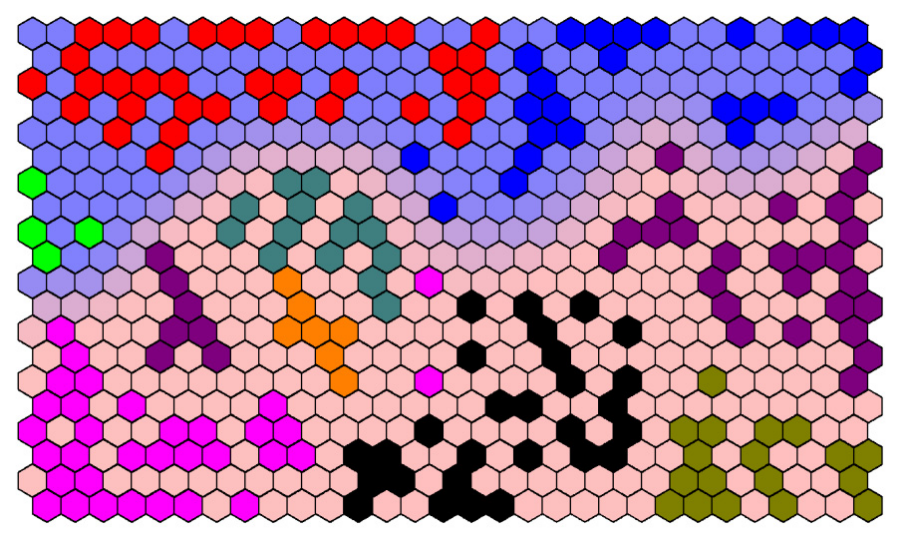
Superimposing different types of information.The BMUs shaded dark blue, red and light green represent amorphous polymers (blue background) whereas the remaining classes represent semi-crystalline polymers (red background).

Different types of structure can be represented on such diagrams. For case study 2, we would classify the samples into amorphous or semi-crystalline or else into one of nine groups. The two types of information can be presented on a single diagram. The background represents the two main types of polymer, whereas the BMUs represent the nine groups, as demonstrated in Figure 6.

### Unified distance matrix

The U matrix (or Unified Distance Matrix) was first described by Ultsch and Siemon [60]. The aim of a U-Matrix is to show the similarity of a unit to its neighbours and hence reveal potential clusters present in the map. If there are classes present in the data, then the border between neighbouring clusters can be interpreted as a class border. The ‘unified distance’ of each unit is calculated as the sum of the similarities between the weight vector of a map unit and the weight vectors of its immediate neighbours. The lower it is the more similar the neighbouring cells are. When going from one class to another, we anticipate that the barrier will be high.

**Figure 7:**
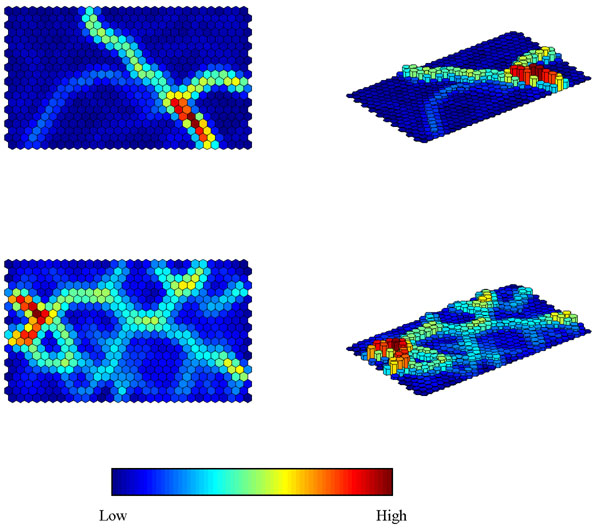
U Matrices for (top) case study 1 and (bottom) case study 2.

A U matrix ideally separates different groups. Figure 7 represents the U matrices for case studies 1 and 2, which can be represented as flat projections or in three dimensions. These should be compared to Figure 5. Consider case study 1. Corn margarine is on the bottom right and can be seen to be quite different to the others. Safflower oil and corn oil are on the left and are seen to be fairly similar. Sometimes the original division of samples into groups is not always reflected in large differences in the corresponding spectra. A close examination of the U matrix for case study 2 suggest that there is some substructure in certain of the polymer groups.

### Component planes

**Figure 8:**
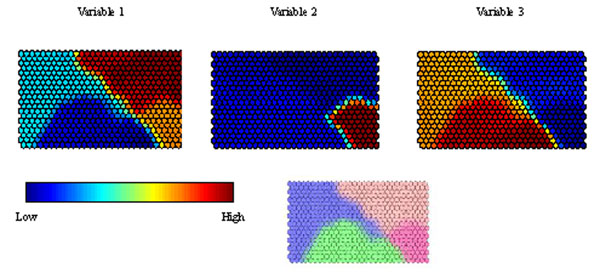
Component planes for three variables (or spectral wavelengths) in case study 1 (top), together with the class map (bottom) for reference.

Each variable has its own component plane. Figure 8 represents component planes for three of the NIR wavelengths. Each has a different profile. Variable 1 has a very high intensity in the top right hand corner, suggesting it is highly diagnostic (or of high intensity) for Olive oil. It has its lowest intensity in the bottom centre group (Safflower oil). Variable 2 is highly intense in corn margarine but of low (or negligible) intensity for all the other groups. Variable 3 is primarily diagnostic of corn margarine and olive oil. This representation is a slice through the weights vector, scaling the highest (or most positive) weight to 1 and the lowest (or least positive) to 0 for each of the variables.

Component planes can be regarded as an analogue of loadings plots, allowing one to determine which variables, if any, are markers (or diagnostic) of a group of samples. There are a number of ways of doing this, but one is to see how similar a component plane of a variable is to its class component plane [56] . A class component plane can be represented by 1s for all cells are closest to BMUs for that class, 0 for cells that are closest to BMUs for another class, and an intermediate value if there are neighbouring BMUs from the class of interest and one or more other classes, rather like the class maps, but in this situation each single class has its own corresponding plane. All component planes for the variables are likewise scaled between 0 and 1. Multiplying the two and summing provides an index for how strongly a variable represents a particular class and can be employed as a form of variable selection or ranking. If there are two classes (or groups) in the data it is possible to subtract the index of one class (B) from that of the other (A). A positive value represents a marker for class A and a negative value for class B. The magnitude of this difference allows ranking of variables according to their perceived relative importance as markers. Where there are more than two groups, the index can be calculated for each of the groups, and subtracted from the index calculated from the groups left out. For example a marker for class A would have a positive value if the index for class A minus the index for all other groups together is positive.

## Supervised SOMs

### Method

SOMs as originally described were primarily for visualisation or exploratory data analysis. However adaptations have been described that allow SOMs to be used in a supervised method, that is for predictive modelling [55,56,61-63].

**Figure 9:**
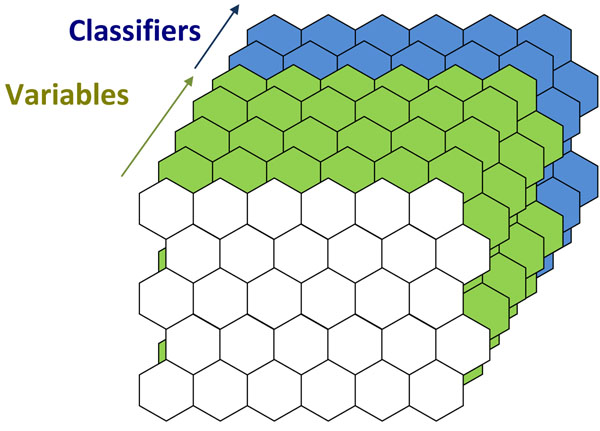
Principles of supervised SOMs.

Figure 9 illustrates the main idea behind supervised SOMs. In addition to the variable component planes, another set of component planes are added that correspond to the class membership. If there are four classes, there are four such planes. These have a value of 1 if a cell corresponds to a sample definitely belonging to a specified class, and 0 if definitely not intermediate values are possible where there is uncertainly. Initially the values are randomly set to a value between 0 and 1. These then are used as extra planes in the training. The relative weight or importance of the variable and class planes can be adjusted. If the class information has a relative weight of 0, the result is the same as an unsupervised map. If the relative weight is very high, the objects are in effect forced into a class structure.

**Figure 10:**
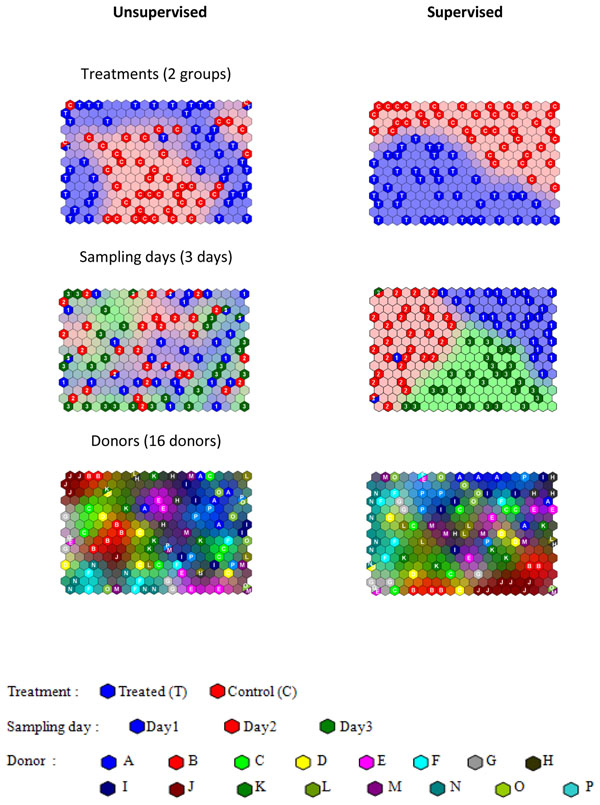
Comparison of Unsupervised and Supervised SOMs for case study 3 (NMR of saliva) trained according to each of the possible three factors or classifiers. Training set BMUs are indicated on the class plots.

When there are many classifiers it is possible to train the map separately for each classifier [56] . For case study 3 (NMR of saliva), the samples can be classified according to whether they were treated with mouthwash or according to sampling day or donor. For unsupervised SOMs these factors are mixed together. The supervised maps are illustrated for each of the three factors in Figure 10 and compared to unsupervised equivalents. Note that the training for each of the factors is quite different, so the samples are positioned in different cells in each of the supervised maps. For the unsupervised SOMs the BMUs are the same, the difference being the shading. Note also that the maps have not been fully forced to provide complete class separation (which can be controlled by adjusting the relative weights of the two types of information).

However a dramatic difference can be seen when comparing the supervised and unsupervised version of the sampling day. In the former the samples are clearly divided into their day of sampling because this has forced the model, but in the latter they are more or less randomly distributed, as this factor has little or no influence, being a dummy factor (Case study 3). Hence supervised SOMs can overfit models. However, an advantage over, for example PLS type approaches is that it is possible to specify the relative importance of the classifier and the measured variable, whereas in PLS they have equal importance.

Supervised SOM representations can, therefore, in themselves, be misleading under certain circumstances, but if correctly employed can be used safely in many situations and as such do provide valuable tools as described below. Note that there is not much literature on how to optimise the relative weights of the class and variable information. However in methods such as PLS, the relative importance of these two types of information is usually fixed so that they are equal, and an advantage of supervised SOMs is that this can be adjusted.

### Determining significant variables

One of the most important uses of supervised SOMs involves determining what variables are important [55,56] for the purpose of defining a class or group of samples, often called marker variables. These may, for example, be characteristic chromatographic peaks or wavelengths. The SOMDI (SOM Discrimination) helps define which variables are significant. The principles are similar to those described in the section on 'Component plans', with a number of additional features. The first is that maps can be forced (or trained) separately for each type of grouping. For case study 3, there are three types of grouping, so an unsupervised SOM would mix these together. A supervised SOM would distinguish these causes of variation and hence can be employed in cases where there several different factors.

**Figure 11:**
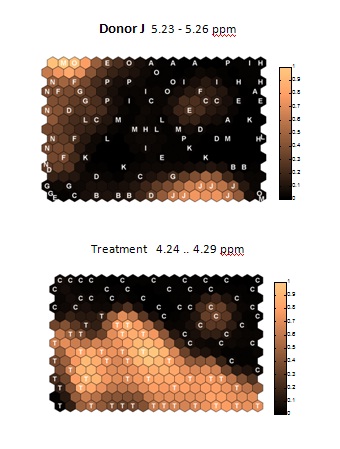
Component planes for supervised SOMs for case study 3, illustrating marker variables for donor J and for treatment. These should be compared to the supervised SOMs in Figure 10. Note light colours indicate a high level of the variable.

Figure 11 illustrates the component planes for two variables, one a marker for treatment in case study 3, and one for donor J in the same case study. These component planes should be compared to the supervised SOMs in Figure 10. For donor J note a dark red cluster of cells in the bottom right of the map, and compare to the light cells (representing high weights) in the component plane in Figure 11. Remember too that the component planes for donors and treatment type are not comparable. This allows different variables to be found. For each class, variables can be ranked according to the similarity of their (supervised) component plane and the supervised map for the corresponding class and factor.

Although a similar exercise could also be performed for the unsupervised map, this only makes sense if the class of interest is the predominant factor and shows grouping in the map.

### Determining the number of significant variables

In this section we describe how to determine which variables are most significant, or are the most likely to be markers, for each class or grouping. However this does not necessarily mean they are significant, it simply ranks variables in order of importance. In case study 3, we expect there to be several strongly significant variables for the treatment type, but none for the sampling day, which is a dummy variable. Yet all variables will be ranked for each type of factor.

There are a number of ways of determining significance. One way [55] is to reform the map many times, from different random starting points. A factor that is significant will remain significant (or a "positive marker") over all the iterations. A variable this is not significant will only randomly appear on the list as a positive marker, and will sometimes appear as a negative marker. If the SOM is reformed 100 times, then a marker that appears to be a positive marker in all 100 iterations can be viewed as having 99% confidence of being a true marker. A marker that is positive some of the time and negative other times is not a stable marker and therefore not considered significant. Of course the more the times the SOM is formed the higher the confidence level. It is recommended to form models around a hundred times, to obtain a 99% confidence level, unless the number of samples is much less than this. Naturally this method requires good computing power. If 10,000 iterations are required to form a SOM, then this is repeated 100 times, 1 million iterations are needed. This can be expedited using parallel processors, such as quadcore or even cluster computers, using for example parallel processing in Matlab using Linux. Although many packages may have been written prior to the widespread advent of parallel processors, it is a simple task to code SOMs into most modern environments using widespread programming tools.

### Predictive modelling

It is possible to perform predictive modelling to determine what class an unknown is a member of. In such circumstances the sample is not part of the original training set, but after training, a test set of samples that are left out [3] can then be assessed. The BMU of each sample in the test set can then be obtained, and the %CC (percentage correctly classified) can be calculated. If the BMU of the test set sample is on the boundary of two classes, then the sample is apportioned to each class, for example, if a BMU for a test set sample is equidistant between the BMU of training set samples from classes A and B, it is assigned as belonging 50% to each of these classes. If this happens a lot, one solution would be to increase the resolution of the map.

Using an independent test set protects against overfitting. By increasing the relative importance of the classifier, apparently excellent separation between groups can be obtained but this is not always meaningful. An example is sampling day in case study 3 (Figure 10). Whereas the %CC of the training set is 92.36%, that for the test set is 38.19%, only slightly above a random model of 33.33% as there are three sampling days [55]. For treatment, the %CC for the training set of 94.72% is reduced to 70.79% in the test set which is well above a random model.

Another problem arises if new (unknown) samples are members of none of the predefined groups. We will show how to deal with this in the next section.

## SOMs in quality control

SOMs have a role in QC (Quality Control) or MSPC (Multivariate Statistical Process Control) [57]. Such problems involve one class classifiers [64]. The NOC (Normal Operating Conditions) samples are a set of samples that are considered "normal", that is of acceptable quality. The aim of MSPC is to determine whether future samples belong to this group and if so with what confidence. If they do not there may be some problem with the process.

A SOM can be obtained from the NOC samples. A problem here is that an unknown sample will always find its place on the map, as there will always be a BMU for any sample, even if the match is not very good. An additional measure, that is not normally taken into account, determining whether a sample is a member of a group or not, is how well the sample fits into the map [57].

**Figure 12:**
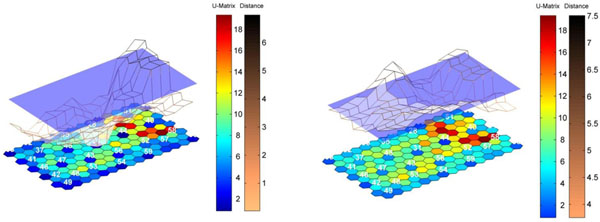
Illustration of SOMs for QC (Quality Control) using case study 4. The distance of a sample to a U matrix of NOC samples is illustrated. On the left is an in-control sample and on the right an out of control sample.

Figure 12 illustrates the principle using case study 4 (the pharmaceutical process). The map (represented here as a U matrix) consists of 30 samples chosen to be the NOC region. There is only one group (or class) of samples under consideration, and in this situation the question is whether an unknown or new sample fits well into the pre-existing group rather than which of a set of pre-existing groups a sample belongs to. The way to do this is to look at the similarity (or distance) of the unknown sample to all cells in the map. If it is far from all the cells then it does not belong to the existing group and as such would be regarded as out of control. If it is close to some of these cells (in other words, similar to some samples from the NOC region) it fits into the group. In the figure a grid is formed of the distances (or dissimilarities) to the map. The left hand diagram represents a sample that fits well into the class model, and so can be regarded as in control, and the right hand a sample that does not fit well and is out of control.

This additional measure can be used in other situations, for example, we may measure the properties of 6 polymers, and want to test whether an unknown sample is a member of the prefedined group or a new group that was not part of the original test set.

## Conclusion

SOMs have a strong potential in chemistry. Although there is a small and growing literature and reports have been available over many years, as yet these techniques are not as widespread as more common methods such as PCA and PLS, probably because many hands-on chemists mainly want to use commercial plug-in packages. Yet SOMs have tremendous flexibility and many of the limitations of the past, such as problems with computer power, are no longer so serious. In analytical chemistry, SOMs can be adapted to specific situations, for example by using supervised SOMs or in Quality Control, and so have a much wider applicability than just visualisation.

## Competing interests

There are no competing interests. The author has no current contracts with or consultancy agreements with the software companies mentioned in this paper and does not advocate or others the use of any specific commercial package.
